# Erratum

**DOI:** 10.1200/JGO.2016.006833

**Published:** 2016-08-10

The meeting abstract by Mantzavinou et al entitled “Implants for Cost-Effective and Accessible Treatment of Gynecologic and Gastrointestinal Cancer Metastasis by Local and Sustained Low-Dose Chemotherapy” (J Glob Oncol 10.1200/JGO.2016.004630), published online in the 4th Annual Symposium on Global Cancer Research supplement, has been updated.

The author order was given incorrectly as Aikaterini Mantzavinou, Michael J. Cima, and Laura Melanie Tanenbaum. The correct author order is Aikaterini Mantzavinou, **Laura Melanie Tanenbaum, and Michael J. Cima**.

This has been corrected as of August 10, 2016.

